# The abundance of the short GATA1 isoform affects megakaryocyte differentiation and leukemic predisposition in mice

**DOI:** 10.1186/s40164-024-00492-9

**Published:** 2024-02-26

**Authors:** Daishi Ishihara, Atsushi Hasegawa, Ikuo Hirano, James Douglas Engel, Masayuki Yamamoto, Ritsuko Shimizu

**Affiliations:** 1 Department of Molecular Hematology, Tohoku University Graduate School of Medicine, 2-1 Seiryo-machi, Aoba-ku, Sendai, 980-8575 Japan; 2 Tohoku Medical Megabank Organization, Tohoku University, Sendai, 980-8575 Japan; 3 Department of Cell and Developmental Biology, University of Michigan Medical School, Ann Arbor, MI 48109 USA

## Abstract

Transcription factor GATA1 controls the delicate balance between proliferation, differentiation and apoptosis in both the erythroid and megakaryocytic lineages. In addition to full-length GATA1, there is an GATA1 isoform, GATA1s, that lacks the amino-terminal transactivation domain. Somatic *GATA1* mutations that lead to the exclusive production of GATA1s appear to be necessary and sufficient for the development of a preleukemic condition called transient myeloproliferative disorder (TMD) in Down syndrome newborns. Subsequent clonal evolution among latent TMD blasts leads to the development of acute megakaryoblastic leukemia (AMKL). We originally established transgenic mice that express only GATA1s, which exhibit hyperproliferation of immature megakaryocytes, thus mimicking human TMD; however, these mice never developed AMKL. Here, we report that transgenic mice expressing moderate levels of GATA1s, i.e., roughly comparable levels to endogenous GATA1, were prone to develop AMKL in young adults. However, when GATA1s is expressed at levels significantly exceeding that of endogenous GATA1, the development of leukemia was restrained in a dose dependent manner. If the transgenic increase of GATA1s in progenitors remains small, GATA1s supports the terminal maturation of megakaryocyte progenitors insufficiently, and consequently the progenitors persisted, leading to an increased probability for acquisition of additional genetic modifications. In contrast, more abundant GATA1s expression compensates for this maturation block, enabling megakaryocytic progenitors to fully differentiate. This study provides evidence for the clinical observation that the abundance of GATA1s correlates well with the progression to AMKL in Down syndrome.

**Supplementary Information:**

The online version contains supplementary material available at 10.1186/s40164-024-00492-9.

## Key points

1. The abundance of GATA1s is a strong prognostic factor of TMD for leukemia by mediating differentiation of megakaryocytes.

2. Persistent TMD blasts not undergoing differentiation are prone to leukemic transformation.

**Supplementary Information:**

The online version contains supplementary material available at 10.1186/s40164-024-00492-9.

To the Editor,

GATA1 is an essential transcription factor for erythroid and megakaryocyte differentiation. GATA1 possesses two transactivation domains (TADs), which locate either in the amino (N)- or carboxyl-terminus [1]. Somatic mutations in the *GATA1* gene, resulting in the production of a shorter variant lacking the N-terminal TAD (GATA1s), are known to induce transient myeloproliferative disorder (TMD) in newborns with Down syndrome [2]. Symptoms of TMD typically regress spontaneously. However, approximately 20% of Down syndrome children with a history of TMD develop genuine acute megakaryoblastic leukemia (AMKL) due to acquisition of additional genetic events [2, 3]. Recent research revealed a significant association between the level of GATA1s protein and the risk of leukemia development [4]. Nevertheless, the molecular mechanisms underlying leukemic transformation of TMD blasts remain largely unknown. Here, for the first time we generated AMKL in mice expressing exclusively GATA1s at multiple abundances.

We employed two independent transgenic mouse lines, ΔNT-H and ΔNT-M, expressing GATA1s under the regulation of G1HRD (*Gata1*-hematopoietic regulatory domain) [5]. In these two mouse lines, the abundance of transgene-derived mRNA in fetal livers was much higher for ΔNT-H, and comparable for ΔNT-M, than the levels of endogenous GATA1 [5]. As a control, we used a transgenic line of mice (G1HRD-G1) expressing wild-type GATA1 under G1HRD control [5]. The human (*GATA1*) and mouse (*Gata1*) genes are located on the X-chromosome. We intercrossed ΔNT-H, ΔNT-M and G1HRD-G1 transgenic male mice with heterozygous females (*Gata1.05*/X), harboring an allele (*Gata1.05*) which expresses only 5% of wild-type GATA1 [6]. We then examined *Gata1*-deficient male (*Gata1.05*/Y) embryos harboring the various GATA1-related transgenes, referred to as ΔNTR-H, ΔNTR-M, and G1R, respectively at embryonic 18.5 days (E18.5). While *Gata1.05*/Y embryos succumbed to lethality by E12.5 due to anemia caused by GATA1 deficiency [6], ΔNTR-H, ΔNTR-M and G1R males were born alive [5].

**Fig. 1:**
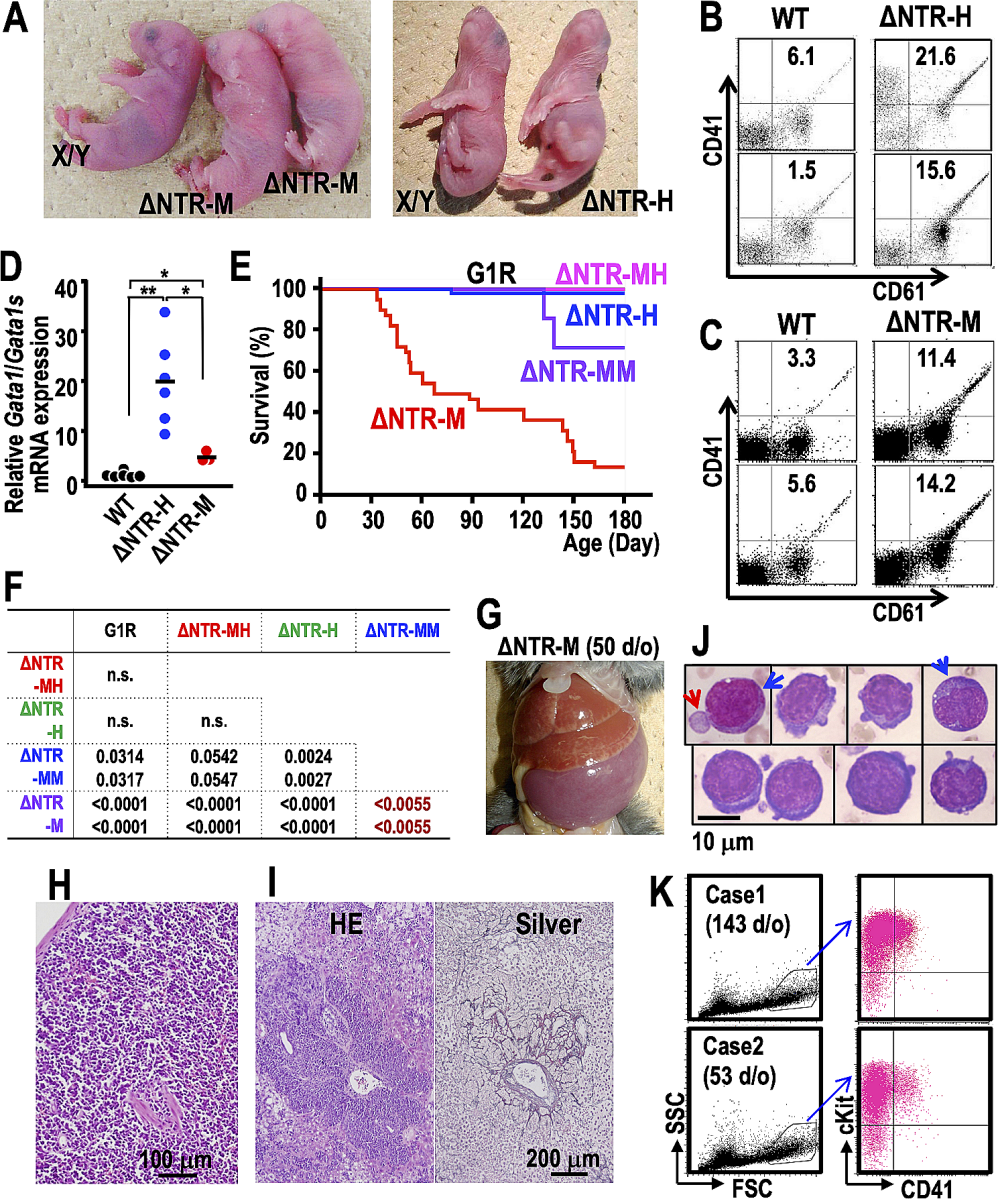
GATA1s transgene-rescued mice develop acute megakaryoblastic leukemias. (**A**) The appearance of ΔNTR-M (left panel) and ΔNTR-H (right panel) embryos at E18.5 and their littermates. (**B**, **C**) Flow cytometric analysis of E18.5 fetal livers from two ΔNTR-H (**B**) and two ΔNTR-M (**C**) mice in comparison to their wild-type littermates. The frequencies of CD41^+^CD61^+^ megakaryocytes in live cells from the spleen are depicted in the panels. (**D**) Expression levels of *Gata1* mRNA in CD41^+^ megakaryocytes. Fetal livers recovered from 3 to 4 wild-type embryos were combined and used as controls. Results are from 6 wild-type control pools, 6 ΔNTR-H, and 3 ΔNTR-M E18.5 embryos. The average value of the wild-type group was set to one, and the average values of ΔNTR-H and ΔNTR-M groups are indicated by black bars. Data were analyzed using the Mann-Whitney U test. *; *P* < 0.05, **; *P* < 0.01. (**E**) Survival curves of 15 G1R (black line), 53 ΔNTR-H (blue line), 39 ΔNTR-M (red line), 12 ΔNTR-MH (magenta line), and 7 ΔNTR-MM (purple line) mice. Black and magenta lines fully overlapped. Note that the early mortality of ΔNTR-M mice was partially or completely suppressed by concomitant expression of GATA1s by the ΔNT-M and ΔNT-H transgenes, respectively (purple and magenta lines versus red line, respectively). Details of mice used in this experiment are in Supplementary Table 1. (**F**) A summary of the Log-rank test (upper row) and Generalized Wilcoxson test (lower row) results for mortality of mice among the indicated groups. Note that ΔNTR-MH survived significantly longer than did ΔNTR-M mice. ΔNTR-MM was significantly rescued from early mortality of the ΔNTR-M, but still markedly prone to suffer from leukemia when compared to G1R, ΔNTR-MH and ΔNTR-H. n.s; not significant. (**G**) Enlarged spleen and liver of a representative ΔNTR-M mouse developing AMKL. (**H**, **I**) Histopathological analyses of spleen (**H**) and liver (**I**) sections with Hematoxylin-Eosin staining (H and left panel of I). Right panel of I is a silver staining of the liver section. Note that destruction of splenic architecture and marked infiltration of blast cells accompanied by increased fibrosis around the liver central vein. (**J**) Blast cells in peripheral blood smear samples of leukemic ΔNTR-M mice. Note that the blasts have basophilic cytoplasm, large nuclei containing several nucleoli and cytoplasmic blebs, likely representing megakaryoblastic leukemia cells. A subset of these blasts have coarse azurophilic granules in the cytoplasm (blue arrows). An abnormally large hypo-granular platelet (red arrow) is observed. (**K**) Flow cytometry analysis of spleen mononuclear cells from a leukemic ΔNTR-M mice. Cells in the abnormal fraction (black polygonal areas in left panels), determined by forward (FSC) and side scatter (SSC) patterns are frequently c-Kit-positive and CD41-dull (right panels). Note that the leukemic cells observed in ΔNTR-M mice harbor megakaryocytic and erythroid immunophenotypes, as seen at high frequency in Down syndrome-related AMKL cases [13]. d/o: days-old

At E18.5, while ΔNTR-H embryos could not be discerned from wild-type littermates as previously demonstrated [7], ΔNTR-M embryos displayed mild anemia (Fig. 1A). Nevertheless, both ΔNTR-M and ΔNTR-H embryos exhibited hyperproliferation of megakaryocytes, consistent with earlier findings (Fig. 1B,C, and Supplementary Fig. 1A) [7]. In line with the findings of flow cytometry analyses, hematoxylin and eosin-stained section of fetal livers from ΔNTR-H and ΔNTR-M mice revealed the accumulation of large megakaryocytes exceeding 10 μm in diameter (Supplementary Fig. 1B). The expressions of GATA1/GATA1s mRNAs in CD41-positive megakaryocytes were approximately 19.7 and 4.7 times higher in ΔNTR-H and ΔNTR-M embryos, respectively, compared to wild-type embryos (Fig. 1D). Therefore, hyperproliferation of megakaryocytes appears to be a consequence of the exclusive expression of GATA1s, and this situation remains unmitigated despite the excessive expression of GATA1s.

Kaplan-Meier analysis (Fig. 1E) revealed that ΔNTR-M mice showed significant early mortality compared with ΔNTR-H and G1R mice. Intriguingly, the early mortality of ΔNTR-M mice was partially and completely restrained by the additional presence of either ΔNT-M or ΔNT-H transgene, respectively. For these rescued mice, we designated *Gata1.05*/Y mice carrying two ΔNT-M transgenes in a homozygous manner as ΔNTR-MM, and *Gata1.05*/Y mice carrying both ΔNT-M and ΔNT-H transgenes in a heterozygous manner as ΔNTR-MH mice. Survival analyses showed significant differences (Fig. 1F). Upon necropsy of nineteen ΔNTR-M mice and one ΔNTR-MM mouse, it was discovered that all of them had severe hepato-splenomegaly (Fig. 1G). The tissue architecture of ΔNTR-M mice revealed infiltrations of aberrant mononuclear cells (Fig. 1H,I and Supplementary Fig. 2). Additionally, marked fibrosis was observed in the livers (Fig. 1I and Supplementary Fig. 2). Peripheral blood films showed the presence of aberrant blasts with cytoplasmic blebs (Fig. 1J and Supplementary Fig. 3). Flow cytometry revealed that the blasts were cKit^+^CD41^dull^ (Fig. 1K). Nude mice transplanted with these blasts consistently developed leukemia that closely resembled the AKML phenotype (Supplementary Fig. 4), indicating that ΔNTR-M mice developed full-blown AMKL. Thus, the GATA1s expression level is a strong prognostic factor of TMD leading to AMKL.

**Fig. 2:**
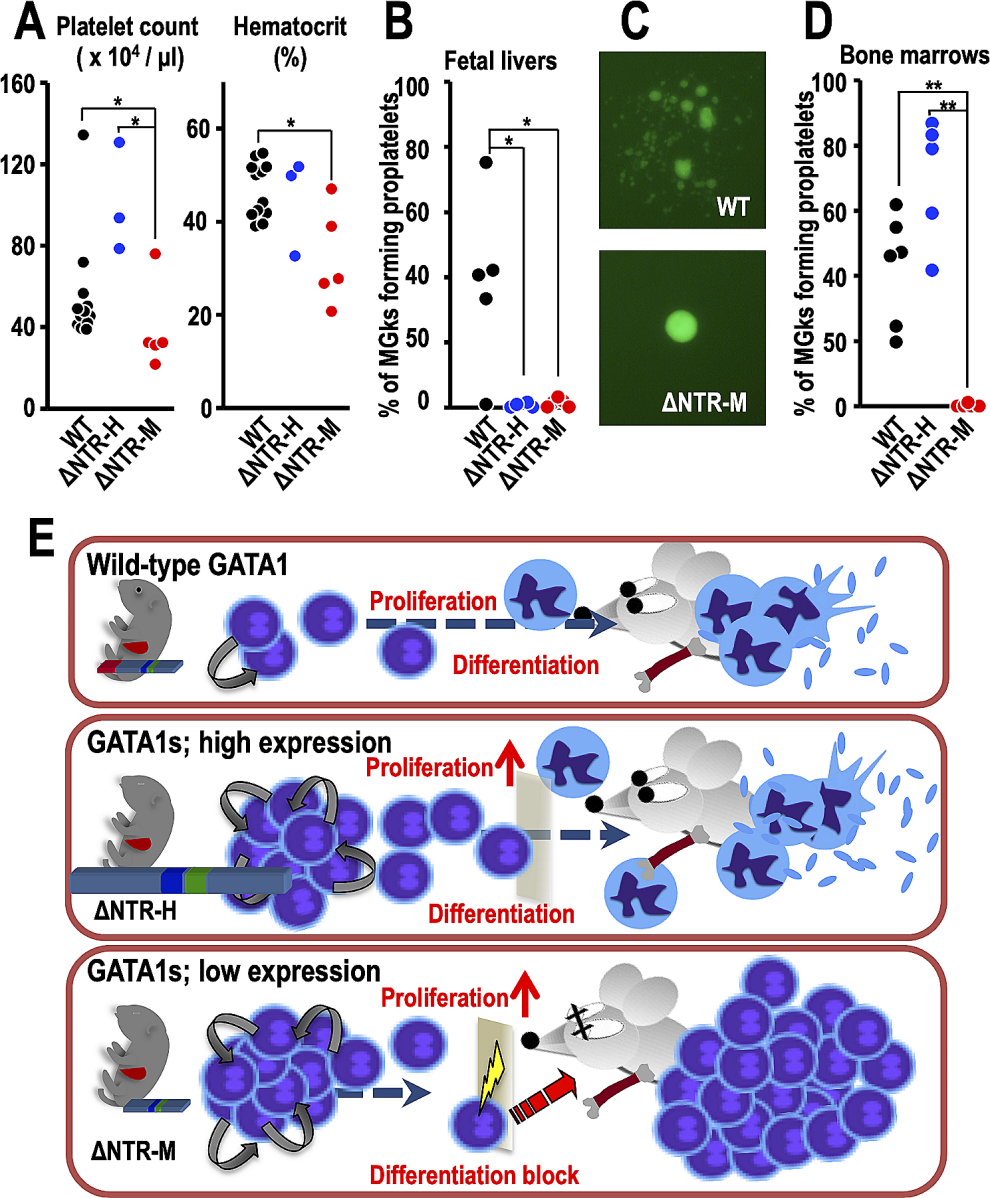
Defects in megakaryocyte maturation due to GATA1s mutation are crucial for leukemia development. (**A**) Platelet counts (left) and hematocrit values (right) of ΔNTR-H and ΔNTR-M embryos at E18.5. (**B**-**D**) Evaluation of proplatelet formation of the rescued mice. Fetal livers at E17.5 or E18.5 (B) and bone marrows at postnatal day 34 (P34) to P47 (**D**) were examined. Fetal livers obtained from 2 to 3 wild-type littermates were combined and used as controls. (**C**) Representative images of megakaryocytes forming proplatelets in wild type (upper panel) and those lacking proplatelet extensions in ΔNTR-M (lower panel) mice. The cytoplasm of megakaryocytes was visualized by green fluorescence protein produced under the regulation of G1HRD [7]. Results from 5 wild-type control samples obtained from each combined sample, 5 ΔNTR-H and 4 ΔNTR-M fetal livers are presented in (**B**) and 6 wild-type, 5 ΔNTR-H and 5ΔNTR-M bone marrow samples are presented in (**D**). The data was analyzed using the Mann-Whitney U test. *; *P* < 0.05, **; *P* < 0.01. (**E**) A model depicting the development of AMKL in ΔNTR-M mice. Wild-type GATA1 supports the balance between proliferation and maturation of megakaryocytes during the process of platelet production (upper panel). Exclusive expression of GATA1s skews immature progenitors toward a proliferation-dominant state (middle and lower panels). However, megakaryocyte progenitors in the bone marrow with more abundant GATA1s expression have the capacity to terminally differentiate (middle panel), while those bearing less abundant GATA1s expression are more likely to remain in an immature stage and harbor an increased chance to acquire additional gene mutations (lower panel)

Intriguingly, while the number of megakaryocytes increased in both lines (ΔNTR-H and ΔNTR-M; Fig. 1B,C), platelet counts were significantly diminished in ΔNTR-M embryos when compared to wild-type (Fig. 2A, left panel). Hematocrit value of ΔNTR-M embryos was significantly lower compared to that of wild-type embryos (Fig. 2A, right panel), which is in good agreement with the anemic appearance of ΔNTR-M embryos (Fig. 1A, left panel). In vitro proplatelet formation assays revealed that, although embryonic megakaryocytes of both ΔNTR-H and ΔNTR-M mice lost the ability to form proplatelets with long filamentous branches (Fig. 2B,C), bone marrow-derived megakaryocytes of ΔNTR-H mice were able to restore proplatelet formation, but those of ΔNTR-M mice were not (Fig. 2D). Thus, embryonic megakaryocytes exclusively expressing GATA1s retain a reduced ability to differentiate and form platelets. However, this defect can be partially compensated after birth if GATA1s is abundant. Similar phenomenon has been observed in induced pluripotent stem cells excursively expressing GATA1s in which differentiation can be altered by the level of GATA1s [8].

To date, two types of leukemias caused by abnormal GATA1 function have been documented [9]. One is erythroleukemia, which occurs in *Gata1*-knockdown female mice (reduced abundance) [10]. The other is AMKL due to GATA1 mutation, leading to a short form of GATA1 (i.e., GATA1s), found in Down syndrome children [2] and in mice as firstly explored here. In the cases of erythroleukemia, immature erythroid progenitors accumulate due to a combination of differentiation arrest and protection from apoptosis [10–12]. These unnatural erythroid progenitors accumulate cancerous changes at a high frequency, leading to the transformation of progenitors into leukemic cells [9]. In the latter cases, AMKL-type leukemogenesis arises from megakaryocytic progenitors that persist in the bone marrow without proper terminal maturation (Fig. 2E). We propose that an essential prognostic determinant of AMKL development is the expression level of GATA1s in TMD blasts, and whether that abundance is adequate to facilitate TMD blast differentiation into terminally matured megakaryocytes.

### Electronic supplementary material

Below is the link to the electronic supplementary material.

Supplementary Material 1

## Data Availability

All data generated or analyzed during this study are included in this published article and its supplementary information file.

## Abbreviations

TADs: Transactivation domains
TMD: Transient myeloproliferative disorder
AMKL: Acute megakaryoblastic leukemia
G1HRD: *Gata1*-hematopoietic regulatory domain
FSC: Forward scatter
SSC: Side scatter
