# Supplementary material for: The abundance of the short GATA1 isoform affects megakaryocyte differentiation and leukemic predisposition in mice
Source: Exp Hematol Oncol. 2024 Feb 26;13:24. doi: 10.1186/s40164-024-00492-9 (PMC10895780; doi:10.1186/s40164-024-00492-9)
Supplement: Supplementary file 1 — Supplementary Material 1 [file 40164_2024_492_MOESM1_ESM.pdf]

# Expression levels of GATA1s isoform affect megakaryocyte differentiation and leukemic predisposition in mice

Daishi Ishihara, Atsushi Hasegawa, Ikuo Hirano, James Douglas Engel,
Masayuki Yamamoto and Ritsuko Shimizu

## List of Supporting Information

Supplementary Fig. 1: Increase of megakaryocytes in ΔNTR-H and ΔNTR-M embryos

Supplementary Fig. 2: Histopathological analyses of liver sections of leukemic ΔNTR-M mice

Supplementary Fig. 3: Peripheral blood smear of a leukemic ΔNTR-M mouse

Supplementary Fig. 4: Autonomous proliferation of leukemic cells in recipient nude mice.

Supplementary Fig. 5: Integration sites of transgenes in the lines of mice expressing GATA1s.

Supplementary Table 1: List of mice used for Kaplan-Meier analysis.

Supplementary Table 2: Primer sequences used for mouse genotyping.

Supplementary Table 3: Primer sequences used for Identification of transgene insertion sites.

Supplementary Table 4: Primer sequences used for qPCR.

Supplementary information: Materials and Methods

**Supplementary Fig. 1**

Increase of megakaryocytes in ΔNTR-H and ΔNTR-M embryos

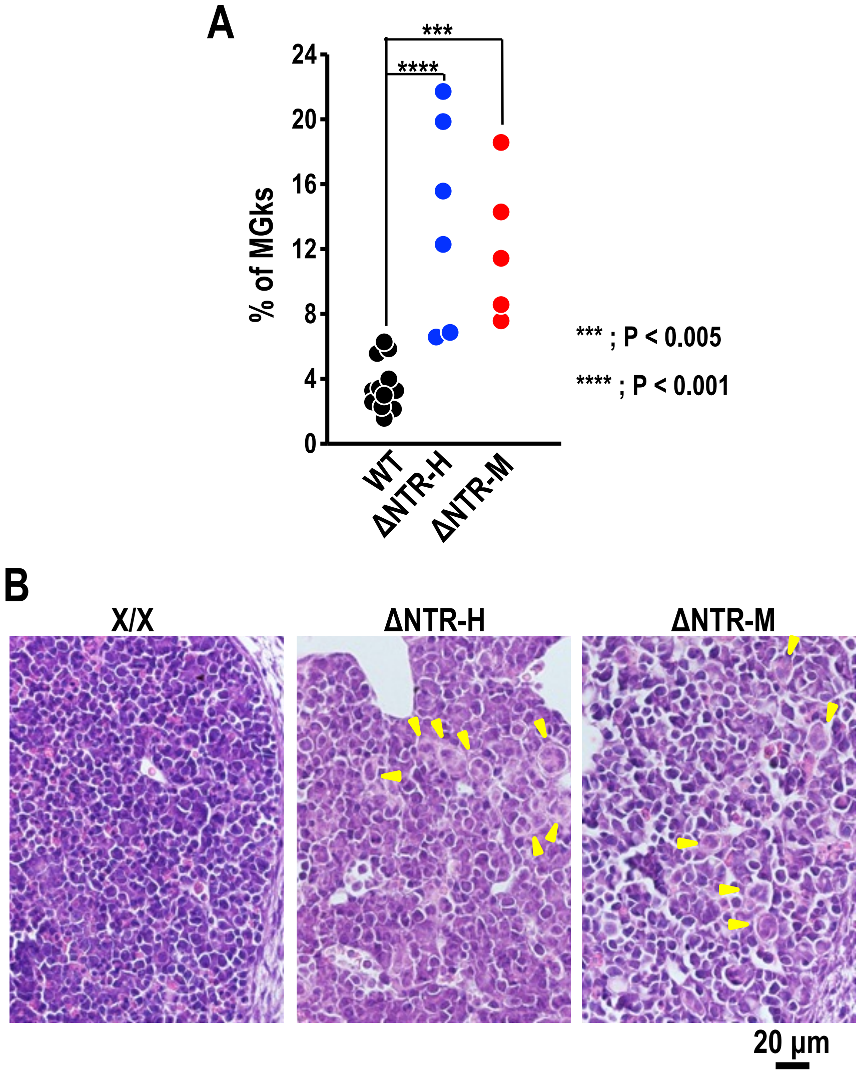


(A) Dot plot depicting the frequencies of CD41+CD61+ megakaryocytes in live cells from the fetal liver at E18.5. (B) Hematoxylin and eosin-stained sections of E12.5 ΔNTR-M and ΔNTR-H livers exhibit accumulation of large cells with a diameter exceeding 10 µm (indicated by yellow allowheads).

**Supplementary Fig. 2**

**Histopathological analyses of liver sections of leukemic ΔNTR-M mice**

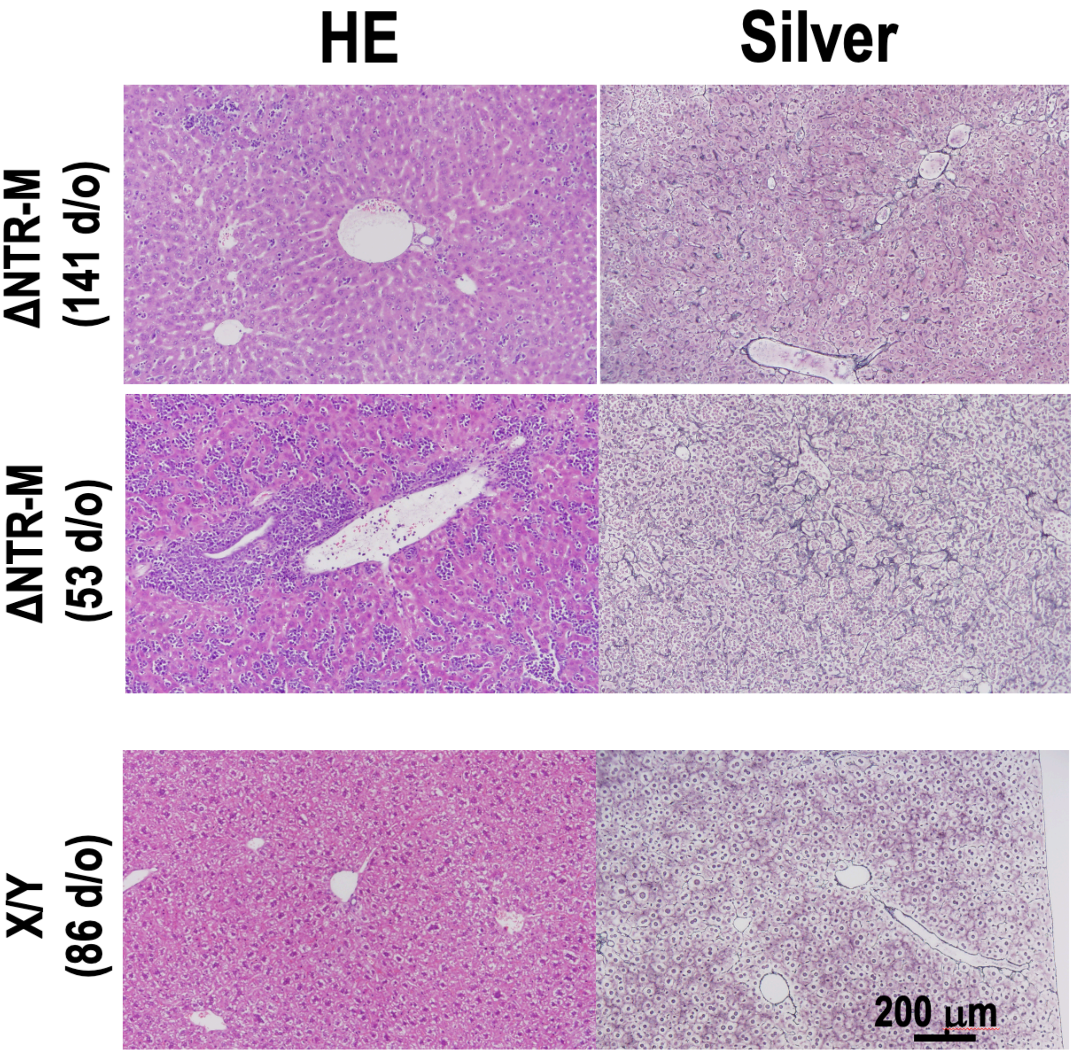


Histopathological analyses on liver sections from two ΔNTR-M mice exhibiting splenomegaly and a control wild-type mouse. Hematoxylin-Eosin staining (left panels) and silver staining (right panels) are shown. d/o: days-old.

**Supplementary Fig. 3**

**Peripheral blood smear of a leukemic ΔNTR-M mouse**

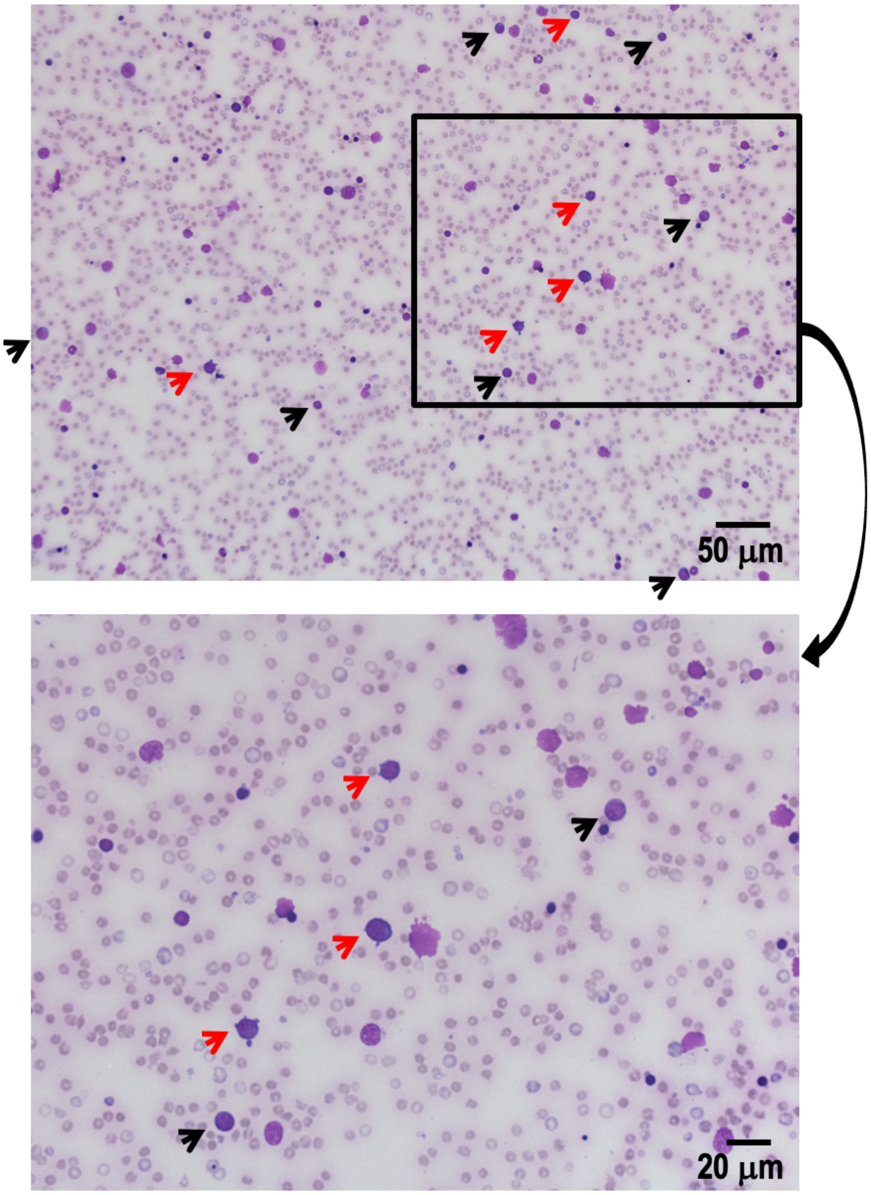


A lower magnification image (upper panel) and an enlarged image (lower panel) of the inset are presented. In these images, multiple blast cells with or without bleb formation are observed, which are indicated by red and black arrows, respectively. Additionally, numerous smudge cells are present, suggesting a hematopoietic neoplastic disease.

**Supplementary Fig. 4**

**Leukemic cells in ΔNTR-M mouse show autonomous proliferation in recipient nude mice.**

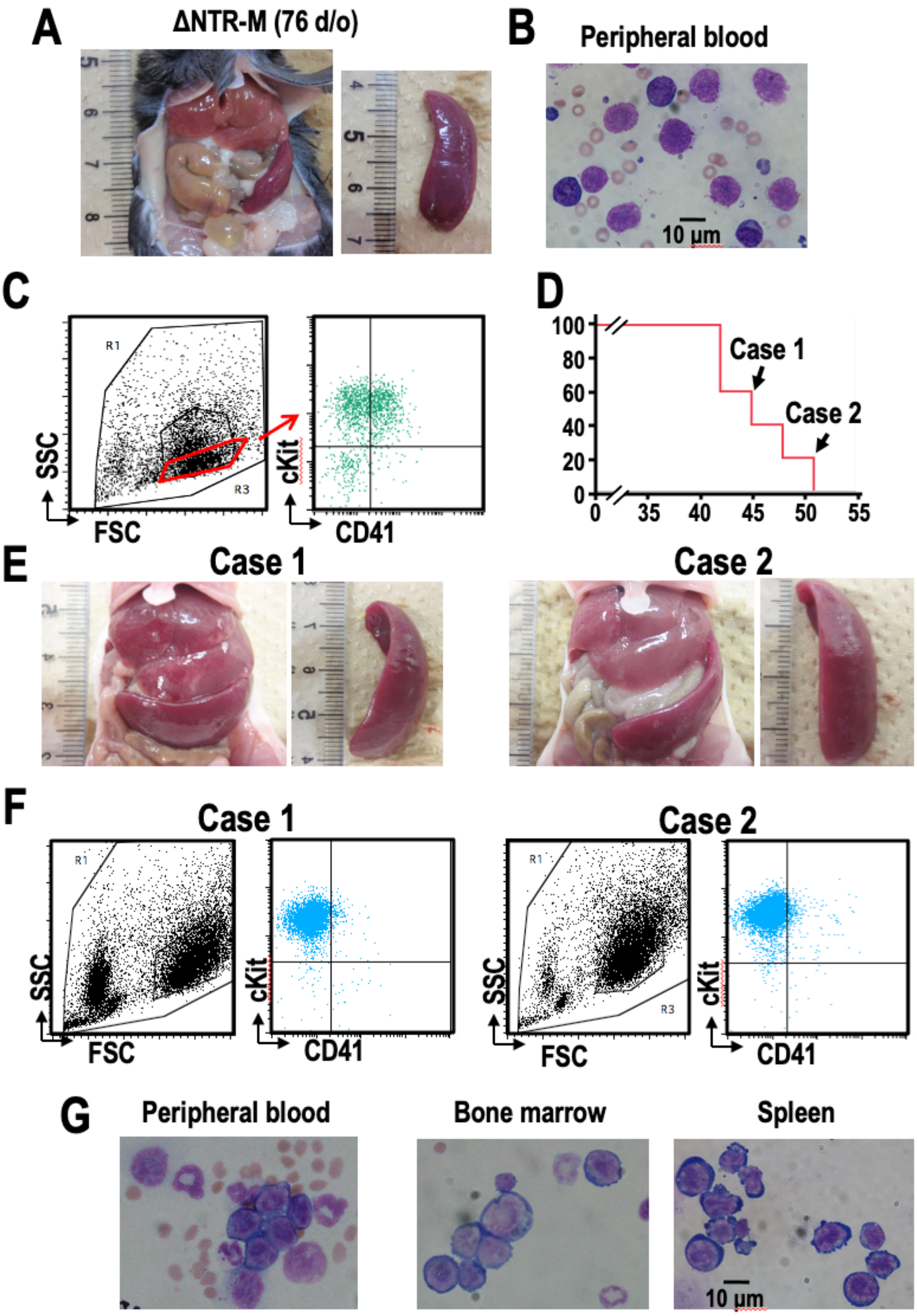

(A-C) A leukemia-developing ΔNTR-M mouse of 76 days-old (d/o) used for the transplantation analysis. Results include the macroscopic appearance (A), May-Grünwald–Giemsa staining of a peripheral blood smear (B), and flow cytometry analysis of the spleen (C). (C) Cells are visualized on FSC versus SSC (left panel), and the abnormal cells gated in the red polygon are c-Kit-positive and CD41-dull (right panel). (D) Kaplan-Meier graph depicting the outcome of five nude mice transplanted with 1 x $10^6$ splenic cells from the leukemia developing ΔNTR-M mouse shown in (A). (E) Representative images from macroscopic analysis of two nude mice at 45 days (Case 1; left) and 52 days (Case 2; right) after transplantation. The spleen, liver, and body weights of mice in Case 1 are 1.97 g, 3.33 g and 22.4 g, respectively, while those in Case 2 are 0.98 g, 1.79 g and 13.5 g, respectively. (F) Flow cytometry analysis of spleen mononuclear cells from the Case 1 nude mouse (left) and that from Case 2 nude mouse (right). Cells within the abnormal fractions, marked by small black polygons in both left panels, exhibited characteristics of c-Kit$^{+}$CD41$^{dull}$, as seen in both right panels. (G) Blast cells in the peripheral blood smear sample (left panel) and cytospin samples from bone marrow (middle panel) and spleen (right panel). These samples were collected from the Case 1 leukemic nude mouse. Note that these leukemic cells in the nude mice exhibit similar characteristics to those observed in the donor ΔNTR-M mouse.

**Supplementary Fig. 5**

**Integration sites of transgenes in the ΔNT-H and ΔNT-M lines.**

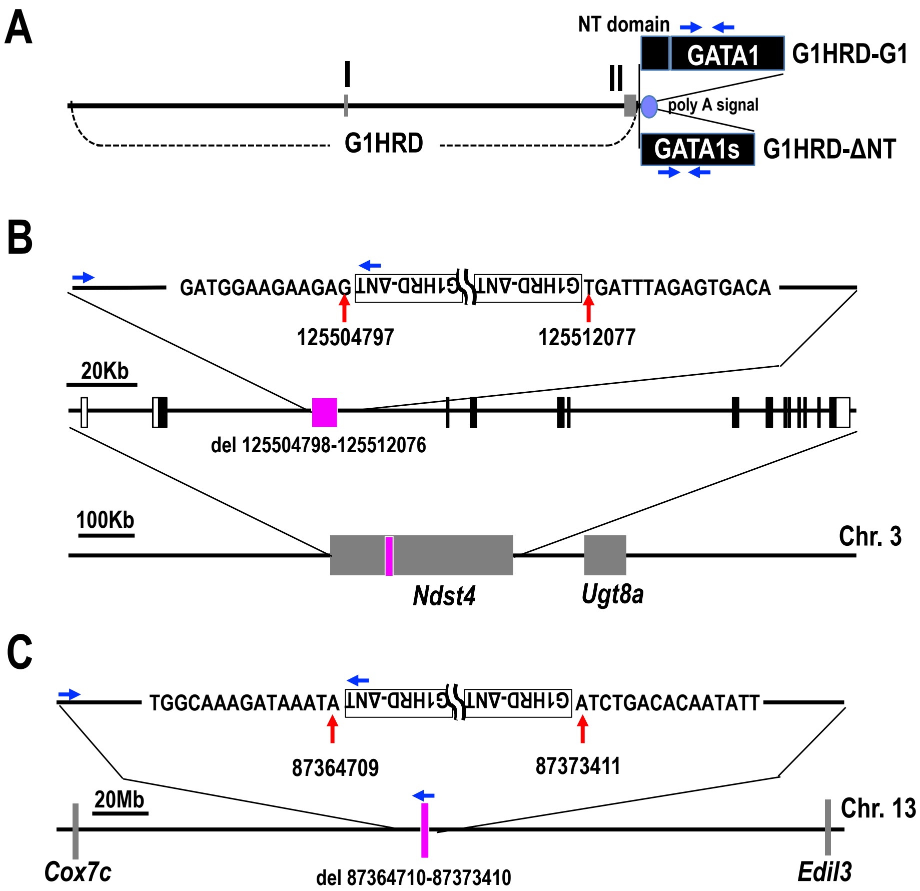


(A) Schematic representation of the G1HRD-G1 and G1HRD-ΔNT transgene constructs. Locations of primers used to identify the transgenes are indicated with blue arrows. (B, C) Schematic diagrams of the transgene-integrated sites on chromosome 3 in the ΔNT-H line of mice (B) and on chromosome 13 in the ΔNT-M line of mice (C). Notably, multiple copies (more than three [6]) of transgenes were integrated into the genomes of ΔNT-H and ΔNT-M transgenic mice. The orientations of the transgenes at both ends are depicted. Genomic position data is referenced from the GRCm38 mouse assembly. Locations of primers used to identify the transgenic and wild-type alleles are denoted with blue arrows. A reverse primer, specific for endogenous chromosome 13 allele, was designed within the sequence that was replaced by transgene in ΔNT-M line. We have determined that the transgene in the ΔNT-H mice is integrated

within the body of the *Ndst4* gene, which may represent a relatively open chromatin structure. In contrast, the transgene in ΔNT-M mice is in an intergenic region approximately 100 Mb away from the neighboring genes, suggesting a probable closed structure. We surmise that the difference in the *Gata1s* expression levels between the two transgenic mouse lines is attributable largely to the insertion site of the transgene.

**Supplementary Table 1**. **List of mice used for experiment shown in Figure 1.**

*: Mice that underwent necropsy

| No. | ID | Age (day) | Genotype | Outcome |
|---|---|---|---|---|
| 1 | 710 | 180 | G1R | Surviving |
| 2 | 957 | 180 | G1R | Surviving |
| 3 | 306 | 180 | G1R | Surviving |
| 4 | 308 | 180 | G1R | Surviving |
| 5 | 309 | 180 | G1R | Surviving |
| 6 | 310 | 180 | G1R | Surviving |
| 7 | 481 | 180 | G1R | Surviving |
| 8 | 487 | 180 | G1R | Surviving |
| 9 | 488 | 180 | G1R | Surviving |
| 10 | 491 | 180 | G1R | Surviving |
| 11 | 432 | 180 | G1R | Surviving |
| 12 | 183 | 180 | G1R | Surviving |
| 13 | 059 | 180 | G1R | Surviving |
| 14 | 738 | 180 | G1R | Surviving |
| 15 | 966 | 180 | G1R | Surviving |
| 16 | 298 | 77 | ΔNTR-H | Unknown |
| 17 | 441 | 180 | ΔNTR-H | Surviving |
| 18 | 496 | 180 | ΔNTR-H | Surviving |
| 19 | 501 | 180 | ΔNTR-H | Surviving |
| 20 | 433 | 180 | ΔNTR-H | Surviving |
| 21 | 434 | 180 | ΔNTR-H | Surviving |
| 22 | 435 | 180 | ΔNTR-H | Surviving |
| 23 | 436 | 180 | ΔNTR-H | Surviving |
| 24 | 350 | 180 | ΔNTR-H | Surviving |
| 25 | 222 | 180 | ΔNTR-H | Surviving |
| 26 | 187 | 180 | ΔNTR-H | Surviving |
| 27 | 724 | 180 | ΔNTR-H | Surviving |
| 28 | 727 | 180 | ΔNTR-H | Surviving |
| 29 | 188 | 180 | ΔNTR-H | Surviving |
| 30 | 191 | 180 | ΔNTR-H | Surviving |
| 31 | 195 | 180 | ΔNTR-H | Surviving |
| 32 | 448 | 180 | ΔNTR-H | Surviving |
| 33 | 351 | 180 | ΔNTR-H | Surviving |
| 34 | 619 | 180 | ΔNTR-H | Surviving |
| 35 | 029 | 180 | ΔNTR-H | Surviving |
| 36 | 030 | 180 | ΔNTR-H | Surviving |
| 37 | 484 | 180 | ΔNTR-H | Surviving |
| 38 | 779 | 180 | ΔNTR-H | Surviving |
| 39 | 223 | 180 | ΔNTR-H | Surviving |
| 40 | 083 | 180 | ΔNTR-H | Surviving |
| 41 | 670 | 180 | ΔNTR-H | Surviving |
| 42 | 672 | 180 | ΔNTR-H | Surviving |

**Supplementary Table 1 (Continued)**

| No. | | ID | Age (day) | Background | Outcome |
|---|---|---|---|---|---|
| 43 | | 673 | 180 | ΔNTR-H | Surviving |
| 44 | | 674 | 180 | ΔNTR-H | Surviving |
| 45 | | 031 | 180 | ΔNTR-H | Surviving |
| 46 | | 061 | 180 | ΔNTR-H | Surviving |
| 47 | | 005 | 180 | ΔNTR-H | Surviving |
| 48 | | 808 | 180 | ΔNTR-H | Surviving |
| 49 | | 470 | 180 | ΔNTR-H | Surviving |
| 50 | | 482 | 180 | ΔNTR-H | Surviving |
| 51 | | 337 | 180 | ΔNTR-H | Surviving |
| 52 | | 653 | 180 | ΔNTR-H | Surviving |
| 53 | | 174 | 180 | ΔNTR-H | Surviving |
| 54 | | 176 | 180 | ΔNTR-H | Surviving |
| 55 | | 165 | 180 | ΔNTR-H | Surviving |
| 56 | | 166 | 180 | ΔNTR-H | Surviving |
| 57 | | 167 | 180 | ΔNTR-H | Surviving |
| 58 | | 198 | 180 | ΔNTR-H | Surviving |
| 59 | | 104 | 180 | ΔNTR-H | Surviving |
| 60 | | 101 | 180 | ΔNTR-H | Surviving |
| 61 | | 102 | 180 | ΔNTR-H | Surviving |
| 62 | | 194 | 180 | ΔNTR-H | Surviving |
| 63 | | 957 | 180 | ΔNTR-H | Surviving |
| 64 | | 491 | 180 | ΔNTR-H | Surviving |
| 65 | | 329 | 180 | ΔNTR-H | Surviving |
| 66 | | 782 | 180 | ΔNTR-H | Surviving |
| 67 | | 964 | 180 | ΔNTR-H | Surviving |
| 68 | | 684 | 180 | ΔNTR-H | Surviving |
| 69 | | 017 | 33 | ΔNTR-M | Unknown |
| 70 | | 199 | 33 | ΔNTR-M | Unknown |
| 71 | | 044 | 35 | ΔNTR-M | Unknown |
| 72 | | 414 | 35 | ΔNTR-M | Unknown |
| 73 | * | 592 | 38 | ΔNTR-M | Leukemia |
| 74 | | 842 | 41 | ΔNTR-M | Unknown |
| 75 | | 864 | 41 | ΔNTR-M | Unknown |
| 76 | | 808 | 45 | ΔNTR-M | Unknown |
| 77 | | 966 | 45 | ΔNTR-M | Unknown |
| 78 | * | 548 | 45 | ΔNTR-M | Leukemia |
| 79 | * | 723 | 45 | ΔNTR-M | Leukemia |
| 80 | * | 387 | 50 | ΔNTR-M | Leukemia |
| 81 | | 728 | 52 | ΔNTR-M | Unknown |
| 82 | | 757 | 52 | ΔNTR-M | Unknown |
| 83 | * | 724 | 53 | ΔNTR-M | Leukemia |
| 84 | * | 756 | 53 | ΔNTR-M | Leukemia |

**Supplementary Table 1 (Continued)**

| No. | | ID | Age (day) | Background | Outcome |
|---|---|---|---|---|---|
| 85 | | 693 | 60 | ΔNTR-M | Unknown |
| 86 | | 721 | 60 | ΔNTR-M | Unknown |
| 87 | | 130 | 67 | ΔNTR-M | Unknown |
| 88 | | 209 | 67 | ΔNTR-M | Unknown |
| 89 | | 384 | 88 | ΔNTR-M | Unknown |
| 90 | * | 071 | 93 | ΔNTR-M | Leukemia |
| 91 | * | 155 | 93 | ΔNTR-M | Leukemia |
| 92 | * | 162 | 120 | ΔNTR-M | Leukemia |
| 93 | * | 209 | 120 | ΔNTR-M | Leukemia |
| 94 | * | 337 | 143 | ΔNTR-M | Leukemia |
| 95 | * | 516 | 143 | ΔNTR-M | Leukemia |
| 96 | * | 494 | 146 | ΔNTR-M | Leukemia |
| 97 | * | 516 | 146 | ΔNTR-M | Leukemia |
| 98 | * | 663 | 149 | ΔNTR-M | Leukemia |
| 99 | * | 792 | 149 | ΔNTR-M | Leukemia |
| 100 | * | 094 | 150 | ΔNTR-M | Leukemia |
| 101 | * | 200 | 150 | ΔNTR-M | Leukemia |
| 102 | * | 396 | 162 | ΔNTR-M | Leukemia |
| 103 | | 117 | 180 | ΔNTR-M | Surviving |
| 104 | | 685 | 180 | ΔNTR-M | Surviving |
| 105 | | 110 | 180 | ΔNTR-M | Surviving |
| 106 | | 738 | 180 | ΔNTR-M | Surviving |
| 107 | | 351 | 180 | ΔNTR-M | Surviving |
| 108 | | 082 | 180 | ΔNTR-MH | Surviving |
| 109 | | 471 | 180 | ΔNTR-MH | Surviving |
| 110 | | 004 | 180 | ΔNTR-MH | Surviving |
| 111 | | 009 | 180 | ΔNTR-MH | Surviving |
| 112 | | 599 | 180 | ΔNTR-MH | Surviving |
| 113 | | 427 | 180 | ΔNTR-MH | Surviving |
| 114 | | 256 | 180 | ΔNTR-MH | Surviving |
| 115 | | 470 | 180 | ΔNTR-MH | Surviving |
| 116 | | 373 | 180 | ΔNTR-MH | Surviving |
| 117 | | 148 | 180 | ΔNTR-MH | Surviving |
| 118 | | 205 | 180 | ΔNTR-MH | Surviving |
| 119 | | 088 | 180 | ΔNTR-MH | Surviving |
| 120 | * | 406 | 132 | ΔNTR-MM | Leukemia |
| 121 | | 431 | 138 | ΔNTR-MM | Unknown |
| 122 | | 657 | 180 | ΔNTR-MM | Surviving |
| 123 | | 355 | 180 | ΔNTR-MM | Surviving |
| 124 | | 071 | 180 | ΔNTR-MM | Surviving |
| 125 | | 020 | 180 | ΔNTR-MM | Surviving |
| 126 | | 151 | 180 | ΔNTR-MM | Surviving |

**Supplementary Table 2. Primer sequences used for mouse genotyping.**

| | | Primer sequence (5’-3’) |
|---|---|---|
| *Gata1.05* allele | F | AAGTATCCATCATGGCTGATGC |
| | R | TAGCCAACGCTATGTCCTGATA |
| Y-chromosome | F | CCTATTGCATGGACTGCAGCTTATG |
| | R | GACTAGACATGTCTTAACATCTGTCC |
| G1HRD-G1 and G1HRD-ΔNT transgene | F | AGATGAATGGTCAGAACCGG |
| | R | AGGCATAAGATGGCTGACAG |
| ΔNT-M transgene | F | GGAGACAGGATCTTCTGTAGC |
| | R | GGGTGATTGGGTTACCTTACTC |
| ΔNT-H transgene | F | CCAGAGGATAGAGAGCAATATC |
| | R | CAAACCACAACTAGAATGCAG |
| Endogenous allele at the ΔNT-M transgene integration site | F | GGAGACAGGATCTTCTGTAGC |
| | R | GTAGTCCTACCTGAGGGACCAG |

**Supplementary Table 3. Primer sequences used for Identification of transgene insertion sites.**

| Primer name | Primer sequence (5'-3') |
|---|---|
| G1HRD outer | GAGTACTCACATGAATCTGAGATGTC |
| G1HRD nested | GAGTATTCTGATCTGTCTACTGAGTGC |
| ΔNT3'UTR outer | GAGACAGGATCTTCTGTAGCTATGTAG |
| ΔNT3'UTR nested | TTTGGACAAACCACAACTAGAATGCAG |

**Supplementary Table 4. Primer sequences used for qPCR.**

| | | Primer sequence (5’-3’) |
|---|---|---|
| *Gata1* | F | CAGAACCGGCCTCTCATCC |
| | R | TAGTGCATTGGGTGCCTGC |
| *Gapdh* | F | GTCGTGGAGTCTACTGGTGTCTT |
| | R | GAGATGATGACCCTTTTGGC |

## Supplementary methods

### Mice

Experimental procedures involving animals are approved by the Institutional Animal Experiment Committee of the Tohoku University. Experiments were carried out in compliance with the Regulation for Animal Experiments in Tohoku University (2015MdA-001, 2018MdA-058, 2018MdA-245, 2019MdA-289). Mice were continuously monitored for survival and were humanely euthanized upon reaching predetermined endpoints. Mice having enlarged spleen over the midline of body at necropsy were defined as those developing leukemia. Hematopoietic indices of embryos were measured by collecting blood after decapitation with surgical scissors and using a Celltac-α autohemocytometer (Nihon Koden). The *Gata1.05* allele was identified through genomic polymerase chain reaction (PCR) using a pair of primers specific to the neomycin resistance gene [6]. *Gata1* transgenes were genotyped via PCR, using a primer set designed to target the region flanking exon 4 and exon 6 of *Gata1* cDNA (Supplementary Fig. 5A). ΔNTR-MH mice were produced by crossing *Gata1.05*/X mice carrying ΔNT-H or ΔNT-M transgene with ΔNT-M or ΔNT-H transgenic male mice, respectively. Specific primer sets were used to identify ΔNT-H and ΔNT-M transgenes (Supplementary Fig. 5B, C). ΔNTR-MM mice were generated by crossing *Gata1.05*/X mice carrying the ΔNT-M transgene with ΔNT-M transgenic male mice. To distinguish ΔNTR-MM from ΔNTR-M, we used a primer set designed to recognize the endogenous allele at the site where the ΔNT-M transgene was incorporated (Supplementary Fig. 5). The primer sequences used for mouse genotyping are shown in Supplementary Table 2.

### Identification of insertion sites of transgenes

Transgene insertion sites were determined using the Universal Genomic Walker Kit from

Clontech Laboratories, following the manufacturer's instruction. In brief, we amplified fragments containing genome-transgene boundary sequences from the transgenic mouse genome through nested PCR. This was achieved using transgene-specific primers in conjunction with adaptor primers provided by the manufacturer. The sequences of transgene-specific primers are shown in Supplementary Table 3. DNA fragments were purified from agarose gel and cloned into a pGEM-T vector (Promega). Subsequently, sequencing of the cloned fragments was carried out using T7 and SP6 primers, employing BigDye Terminator v3.1 Cycle Sequencing Kit and ABI PRISM 3100 Genetic Analyzer (Applied Biosystems). After excluding fragment sequences originating from the fusion of two transgenes, sequence data were analyzed through the National Center for Biotechnology Information (https://www.ncbi.nlm.nih.gov) and the DNA Data Bank of Japan (https://www.ddbj.nig.ac.jp/index-e.html) websites. Schematic illustrations depicting the genomic regions surrounding the transgenes are shown in Supplementary Fig. 5.

**Flow cytometry analysis**

Mononuclear cells collected from fetal liver and spleen were suspended in phosphate-buffered saline (PBS) containing 2% fetal bovine serum (FBS). Fetal liver cells were labeled with fluorescein isothiocyanate (FITC)-conjugated anti-CD41 (eBioscience) and phycoerythrin (PE)-conjugated anti-CD61 (eBioscience) antibodies. Spleen cells were labeled with FITC-conjugated anti-CD41, PE-conjugated anti-CD71 (BioLegend) and allophycocyanin conjugated anti-cKit (BD Biosciences) antibodies. After two washes with PBS, the cells were resuspended in PBS containing 2% FBS and analyzed using FACS Calibur™ (Becton Dickinson Biosciences). Dead cells were excluded by propidium iodide.

**RNA extraction and quantitative real-time PCR**

CD41-positive cells were isolated using biotin anti-CD41 antibody (eBioscience) and

streptavidin-conjugated beads (Invitrogen). Total RNA from the CD41-positive cells was isolated using ISOGEN (NIPPON GENE). Subsequently, first-strand cDNA was synthesized using ReverTra Ace (TOYOBO). Quantitative real-time PCR was conducted utilizing the ABI PRISM 7300 sequence detector system and StepOnePlus Real-Time PCR System (Applied Biosystems) along with THUNDERBIRD SYBR qPCR Mix (TOYOBO). The data were normalized to the *Gapdh* mRNA level. The primer sequences employed for the expression analysis are provided in Supplementary Table 4.

**Proplatelet formation assay**

Mononuclear cells were suspended in CATCH-medium (doi: 10.1083/jcb.69.1.159) and overlaid on a discontinuous BSA gradient (2%/3%/4%/16% in CATCH-medium). After 1 hour, the upper half of the gradient was combined and washed with CATCH-medium. Subsequently, cells were resuspended in 2-ml IMDM-medium containing Nutridoma-SP (Behringer-Mannheim). After a 24 hour-incubation at 37ºC, the frequencies of megakaryocytes forming proplatelets were assessed.

**Histological and cytological analysis and microscope**

Cytospin samples of bone marrow and spleen cells were prepared using a cytocentrifuge (Thermo Fisher Scientific). Peripheral blood smear samples and cytospin samples were stained with May–Grünwald staining solution (Merck) and Giemsa staining solution (Merck). Formalin-fixed tissue sections were stained with Hematoxylin-eosin and Watanabe's silver impregnation method. Images of mice were monitored by μTOUGH-8000 (Olympus). Images of tissues were captured using a BX51 microscope (Olympus) and images of cells expressing GFP were captured using MZFLIII stereomicroscopes (Leica), and were photographed using a DP73 CCD-camera (Olympus).

**Statistical analysis**

All statistical analyses were done using JMP software (SAS Institute Inc.). Survival rates were analyzed using the Kaplan-Meier method. The data was analyzed using a Mann-Whitney U test. Mortality of various mouse groups were compared using the Log-rank test and Generalized Wilcoxson test.
